# Water-Borne ZnO/Acrylic Nanocoating: Fabrication, Characterization, and Properties

**DOI:** 10.3390/polym13050717

**Published:** 2021-02-26

**Authors:** Tien Viet Vu, Thien Vuong Nguyen, Mohammad Tabish, Sehrish Ibrahim, Thi Huong Thuy Hoang, Ram K. Gupta, Thi My Linh Dang, Tuan Anh Nguyen, Ghulam Yasin

**Affiliations:** 1 Faculty of Chemical Technology, Hanoi University of Industry, Bac Tu Liem, Hanoi 143510, Vietnam; vuviet.tphn@gmail.com; 2 Institute for Tropical Technology, Vietnam Academy of Science and Technology, Hanoi 122300, Vietnam; dangthimylinh.lhda58@gmail.com; 3 Vietnam Academy of Science and Technology, Graduate University of Science and Technology, 18 Hoang Quoc Viet, CauGiay, Hanoi 122300, Vietnam; 4 State Key Laboratory of Chemical Resource Engineering, College of Materials Science and Engineering, Beijing University of Chemical Technology, Beijing 100029, China; tabish.5000@buct.edu.cn; 5 Department of Zoology, University of Education, Lahore, Punjab 54770, Pakistan; sehrish.ibrahim@yahoo.com; 6 Hong Duc University, 565 Quang Trung, Dong Ve, Thanh Hoa 4000, Thanh Hoa Province, Vietnam; hhthuyhd77@gmail.com; 7 Kansas Polymer Research Center, Department of Chemistry, Pittsburg State University, Pittsburg, KS 66762, USA; 8 Institute for Advanced Study, College of Physics and Optoelectronic Engineering, Shenzhen University, Shenzhen 518060, China

**Keywords:** acrylic polymer, micro-ZnO, nano-ZnO, nanocomposite coating, photocatalytic, weathering aging, self-cleaning

## Abstract

This work aims to explore how ZnO nanoparticles enhance the mechanical, photoaging, and self cleaning properties of water borne acrylic coating. Micro/nano ZnO particles (at 2 wt.% of total solid resin) were dispersed into the acrylic polymer matrices using ultrasonication to understand the effect of the size of the coating properties. The effect of ZnO particles on the properties of composite coatings (25 µm of thick) have been evaluated through various tests, such as abrasion measurement, ultraviolet/condensation (UV/CON) weathering aging, and methylene blue self cleaning. Experimental data indicated that the incorporation of ZnO particles enhanced both abrasion resistance and methylene blue removal efficiency of the water borne acrylic coatings, with nano ZnO particles being the best. However, the weathering degradation of nanocomposite coatings was more severe as compared to the coating with micro ZnO (at the same ZnO content).

## 1. Introduction

Water-borne acrylic paints are widely used to protect and decorate architectural constructions, since they have many advantages, for instance, a low percentage of volatile organic compounds, easy operation, cost-saving, good weathering resistance [1,2,3]. However, the drawback of this kind of paint is that it is prone to mold growth and dirt clinging. To address this problem, the manufacturers usually add some additives to paint formulations, such as Rocima^TM^ 363, Rocima^TM^ 623, etc. These chemicals are harmful to humans and the environment. Hence, finding alternatives to these toxic substances is considered significant for the interest of researchers and socio-economic development [4,5,6,7,8,9,10].

In recent decades, inorganic nanoparticles have been considered as the essential additives to enhance the properties or provide new/multifunctional properties to organic and various nanocoatings [11,12,13,14,15,16,17]. Nanoparticles have attracted considerable research interest due to their unique properties, arising from the quantum confinement effect and very large surface-to-volume ratios. Similar to other semiconductor oxides, like TiO_2_, ZnO could exhibit photocatalytic activity when used in the coating. However, most published works only focused on the micro-sized ZnO particles, with a very limited photocatalytic behavior in the coating system. In the case of the nanocomposite coating, their antimicrobial activity and self-cleaning ability could be achieved, with nano-ZnO being the most promising and effective photocatalyst [18,19,20,21]. As reported, after absorbing UV radiation, an electron of ZnO at the valence band jumps into the conduction band, creating an h^+^ hole [22]. Then, the e^−^/h^+^ pairs move to the surface of the particles, where they can recombine to each other or react with oxygen and water to form the ^•^OH radicals.

The newly generated ^•^OH radicals initiate the decomposition reactions of bacteria and dirt growing on the surface or inside the coatings. Concurrently, these ^•^OH radicals can play a role in promoting the aging of the coatings [19,23]. Under the action of weathering factors, outdoor coatings are significantly degraded [24,25,26]. The supplement of additives can increase or reduce the weathering resistance of the coatings, depending on their characteristics [20,27,28,29,30,31].

In this study, the impact of ZnO particles on the properties of composite coatings has been evaluated through various tests, such as the falling sand abrasion, IR, UV–vis, FE-SEM analysis, weight loss, and the self-cleaning function for methylene blue removal.

## 2. Materials and Methods

### 2.1. Materials

Dispersion acrylic polymer PLEXTOL R 4152 with a solid content of 49 ± 1 wt.% was received from Synthomer (Essex, UK). Texanol (2,2,4-trimethyl-1,3-pentanediol monoisobutyrate) was obtained from the Dow Chemical Company (Bangkok, Thailand). Nano-ZnO particles (<100 nm; ≥99.0%) and micro-ZnO particles (>10 µm; ≥99.0%) were supplied by Sigma Aldrich (Singapore).

### 2.2. Coating Fabrication

**Scheme 1:**
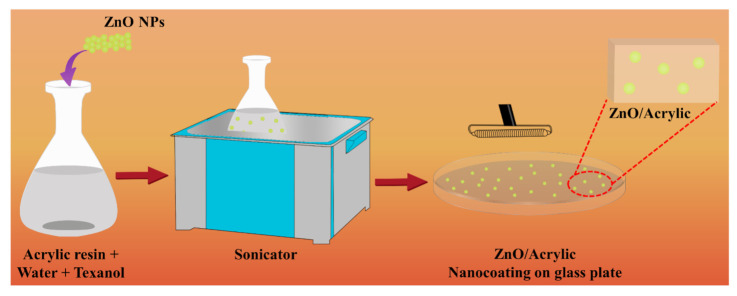
Schematic illustration of the ZnO/Acrylic nanocoating fabrication.

Texanol (3 wt.%) was used as a coalescing agent (Dow Chemical Company, Bangkok, Thailand). The used content of nano-ZnO or micro-ZnO was 2 wt.% (according to the optimal content of nanoparticles in a coating formulation based on our previously published papers [19,32]). The distilled water content was 20 wt.% (by weight on total solid resin). These paint formulations were prepared by the ultrasonic vibration method in a TPC 25 bath (Bronschhofen, Switzerland) for 2 h to ensure that the ZnO nanoparticles were dispersed the best in the dispersion acrylic polymer system. After that, they were coated on the glass plates using a Quadruple Film Applicator (Erichsen model 360, Hemer, Germany) to obtain paint films with a thickness of approximately 25 µm (see Scheme 1). For IR and UV–vis analysis, the dried paint films were removed and attached to aluminum windows.

### 2.3. Coating Characterization

Determination of abrasion resistance: The abrasion resistance of the paint samples was examined by previously published methods [33]. According to the standard ASTM D968, the abrasion test was performed using the sand falling methods. The abrasion tester and silica sand were utilized, and the abrasion resistance (A, litres per mil) was calculated with help of Equation (1).

*A_volume_* = *V/T* (1)

where *V* is the volume of abrasive (L) and *T* is the coating thickness (mils).

Morphology analysis: The dispersion of nano-ZnO and micro-ZnO particles in the coatings was analyzed by a scanning electron microscope S-4800 (FE-SEM, Hitachi, Tokyo, Japan).

IR analysis: The chemical changes of the coatings during the aging process were studied by IR analysis in an FT-IR spectrometer (NEXUS 670 from Nicolet, Waltham, MA, USA). The quantitative analysis of the changes in the functional groups was performed following the reported method [20]. By making the ratio of the IR absorbance at the corresponding wavenumber of the sample before (*D_o_*) and after (*D_t_*) aging cycles, the remaining functional groups were determined, expressed in Equation (2):

*Remaining group(%)* = *(D_t_/D_o_)* × 100 (2)

UV–vis analysis: The UV–vis spectra of the initial coatings as well as the coatings covered by methylene blue before and after 12 h of UV radiation exposure were determined in a UV–vis spectrophotometer (GBC, CINTRA 40, Austin, TX, USA).

Weight loss: The changes in weight of the coatings impacted by the accelerated weathering environment were measured as presented in our previous work [34]. The weight loss (Δ*m_t_*) of the coatings after accelerated WT was determined as the difference between the weights of the samples (dried in a vacuum oven at 50 °C until the constant weight) before (*m_o_*) and after (*m_t_*) the aging process, by Equation (3):

*Δm_t_=[(m_o_‒m_t_)/m_o_]* × 100 (3)

### 2.4. Weathering Aging Test

The coatings for the weathering aging test were exposed to the radiation of UVA—340 fluorescent lamps in ultraviolet/condensation (UV/CON) equipment (model UC-327-2, Chicago, IL, USA), according to the standards ASTM D-4587-05. Each test cycle consisted of 8 h of UV exposure at 60 °C and 4 h of condensate water exposure at 50 °C.

### 2.5. Self-Cleaning Test

The methylene blue solution (0.0162 g of methylene blue dissolved in 20 mL of ethanol) was prepared as the published work [5]. Then, this solution was coated on the paint films by a Quadruple Film Applicator (Erichsen model 360, Hemer, Germany) using a slot of 30 µm. After being dried, the samples were exposed to UV light in the absence of condensation water to make sure that methylene blue was not washed out during the test.

## 3. Results and Discussion

### 3.1. Effect of ZnO Particles on the Mechanical Property of a Water-Borne Acrylic Coating

**Figure 1:**
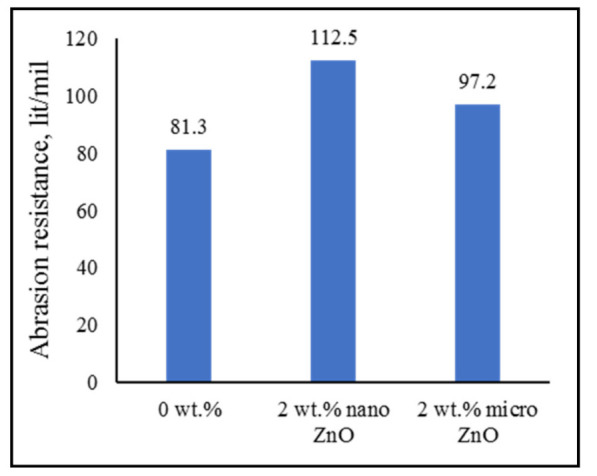
Values of abrasion resistance for the water-borne acrylic coating with or without ZnO particles.

Figure 1 shows the results of the falling sand abrasion test of the coatings which did not consist of ZnO particles, containing 2 wt.% nano-ZnO and 2 wt.% micro-ZnO, respectively.

**Figure 2:**
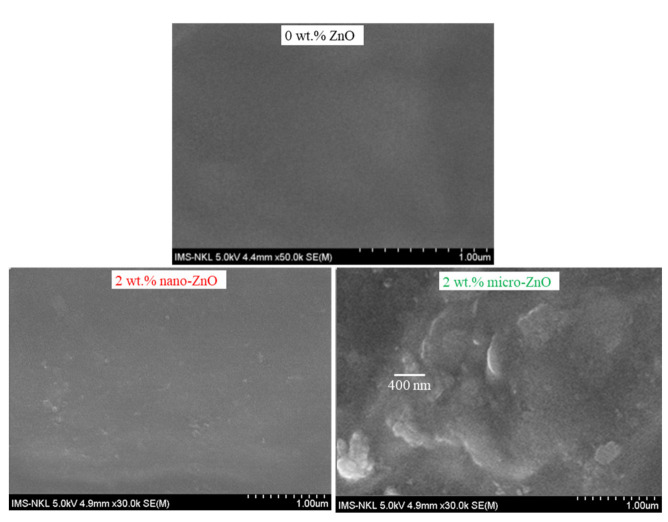
Morphology of acrylic coatings without (0 wt.% ZnO) and with ZnO particles (2 wt.% nano-ZnO and 2 wt.% micro-ZnO).

**Figure 3:**
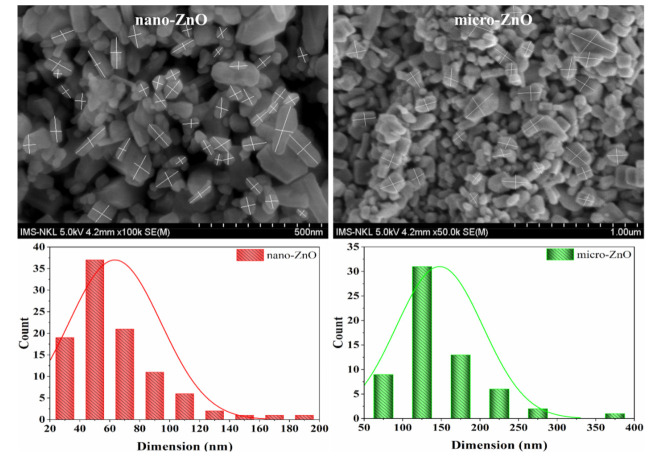
Marked SEM micrograph of the nano-ZnO and micro-ZnO particles for size distribution and their respective particle size distribution histograms.

As can be seen from this figure, the addition of 2 wt.% nano-ZnO enhanced the abrasion resistance of the coating from 81.3 lit/mil to 112.5 lit/mil (corresponding to 38.4%), whilst the supplement of 2 wt.% micro-ZnO only increased from 81.3 up to 97.2 lit/mil (equivalent to 19.5%). The enhancement of abrasion resistance when adding ZnO nanoparticles could be attributed to the polymeric bond formed around the hard ZnO particles [35]. For further explanation, the morphology of the coatings with or without the presence of ZnO particles is presented in Figure 2, while the morphology of the micro-ZnO and nano-ZnO and their respective particle size distribution histogram are shown in Figure 3, which confirms the nano-ZnO particles had the particle size of ≤100 nm, but the micro-ZnO had a particle size in the range of 100–300 nm. Further, the SEM images of nano-ZnO at different magnifications are shown in Figure S1: Supplementary Materials. As can be seen in Figure 2, without ZnO, the neat coating has a homogeneous morphology. In the case of nano-ZnO, the coating shows no sign of nanoparticle agglomeration, leading to the dense structure of the nanocomposite coating, whereas some agglomeration of microparticles is observed when micro-ZnO is added to the coating matrix. As a result, the nanocomposite coating exhibits a higher abrasion resistance.

### 3.2. Effect of ZnO Particles on the Accelerated Weathering Aging of Water-Borne Acrylic Coating

**Figure 4:**
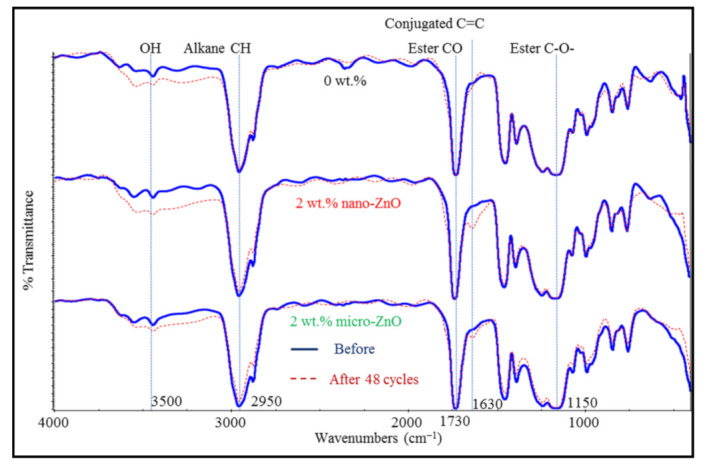
IR spectra of the coatings without ZnO particles (0 wt.%), with 2 wt.% nano-ZnO and 2 wt.% micro-ZnO before and after 48 cycles of UV/CON aging.

**Figure 5:**
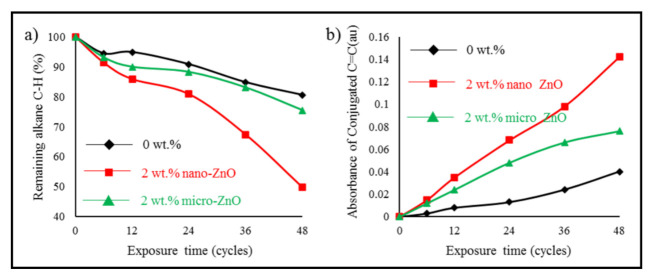
(**a**) Changes of alkane C-H and (**b**) conjugated C=C groups in the coatings with (2 wt.%) or without (0 wt.%) ZnO particles during the UV/CON aging.

IR spectra of the coatings without ZnO particles, with 2 wt.% nano-ZnO, and with 2 wt.% micro-ZnO before and after aging (Figure 4) show that under the effect of UV/CON accelerated weathering factors, the peaks at 2950 cm^−1^ and 1150 cm^−1^ characterizing the groups of alkane C‒H and ester CO in the coatings were reduced. In contrast, the peaks at 3500 cm^−1^ and 1630 cm^−1^ characteristic of the groups of OH and C=C−C=O (conjugated double bond) increased. Among them, the changes of absorbances characterizing the alkane C‒H and C=C−C=O groups are the most obvious rule, so they were chosen to study the changes in the chemical structure of the coatings during aging. The results of the quantitative change analysis of alkane C‒H groups and conjugated double bonds of the corresponding coatings, mentioned above, during the UV/CON aging process are illustrated in Figure 5.

As shown in Figure 4 and Figure 5, under the UV/CON accelerated weathering, the peaks at 2950 cm^−1^ and 1150 cm^−1^ characterizing the stretching of alkane C‒H and ester C‒O‒ in the coatings reinforced by 2 wt.% nano-ZnO decreased more strongly than those of the coating containing 2 wt.% micro-ZnO. In contrast, the peaks at 3500 cm^−1^ and 1630 cm^−1^ representing the stretching of O‒H and C=C‒C=O conjugated double bonds of the coating with nano-ZnO increased significantly compared to the coating with ZnO particles in micro size. In the coatings which do not contain ZnO particles, the alkane C‒H and ester C‒O‒ groups decreased, while the OH and C=C‒C=O groups increased less than those in the coatings containing ZnO particles. After 48 testing cycles, the contents of alkane C‒H groups remaining in the coatings without ZnO, with 2 wt.% nano-ZnO, and with 2 wt.% micro-ZnO were 80.5%, 49.7%, and 75.5%, respectively.

These obtained data suggested that the ZnO particles play an important role as photocatalysts in promoting the aging process of coatings under UV irradiation, with the nano-ZnO being more severe. To prove this point, the UV–vis spectra of the three coating samples, such as neat coating (0 wt.%) and composite/nanocomposite (2 wt.%) coatings, have been analyzed and presented in Figure 5. As seen in Figure 5, the absorbance at 375 nm characterizing the presence of ZnO particles in the coating is much higher for nano-ZnO than that for micro-ZnO. This result indicated that the nano-ZnO absorbs much more UV radiation than micro-ZnO, and thus the photocatalytic behavior could be observed more clearly in the nanocomposite coating. The reason why the photocatalytic activity was much stronger with the presence of nano-ZnO in the coating is the “quantum confinement effect” at the nanoscale (discretization of energy levels), whereas, like bulk matter, the energy bands in micro-ZnO could be formed by merging adjacent energy levels.

**Figure 6:**
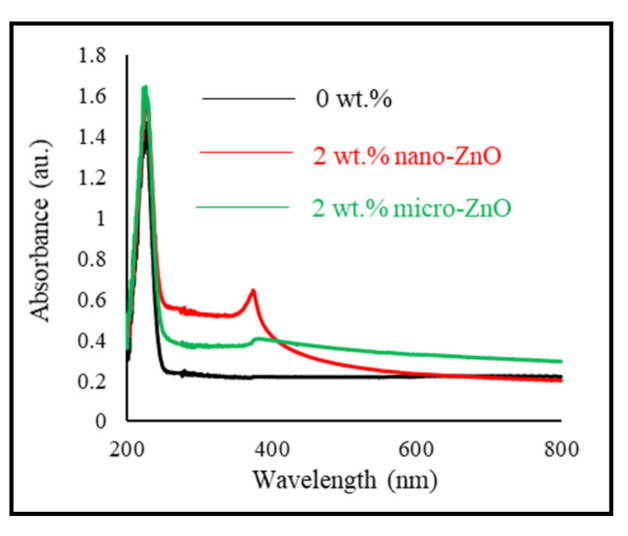
UV–vis spectra of the coatings with (2 wt.%) or without (0 wt.%) ZnO particles.

**Figure 7:**
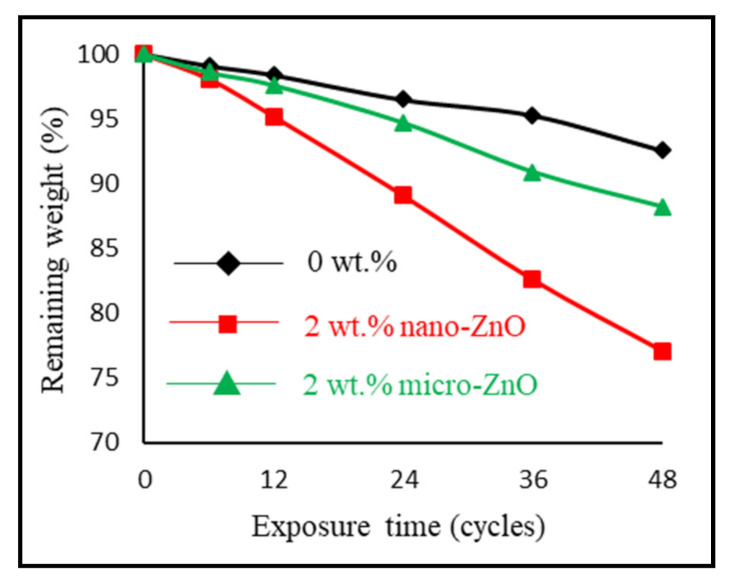
Weight changes of coatings with (2 wt.%) or without (0 wt.%) ZnO particles, during the UV/CON aging.

This finding is consistent with the study of Seentrakoon et al. [36] on the roles of TiO_2_ in the nano and micro scales. It has been explained that the nanoparticles have a much greater specific surface area than the microparticles. Figure 6 also indicates that the coating containing ZnO nanoparticles is more transparent in the visible light region than that consisting of ZnO microparticles with the same content. Figure 7 shows the influence of ZnO particles on the changes in weight of coatings during the aging process. As shown in this figure, during the aging process, the coatings with ZnO particles (nano-ZnO/micro-ZnO) have a higher value of weight loss than the neat coating, with 2 wt.% nano-ZnO being the greatest. After 48 cycles, the weight of the coatings without ZnO particles, with 2 wt.% nano-ZnO, and 2 wt.% micro-ZnO remained 92.6%, 77.1%, and 88.2%, respectively. This shows that during the aging process, the bonds in the polymer chain can be broken by photolysis, photooxidation, photocatalytic, and hydrolysis reactions, forming smaller substances. These compounds can be evaporated or washed out in the presence of condensate water. Due to the strongest photocatalytic activity of ZnO nanoparticles, the nanocomposite has the highest weight loss rate.

### 3.3. Self-Cleaning Performance of Water-Borne Acrylic Coating Containing ZnO Particles

**Figure 8:**
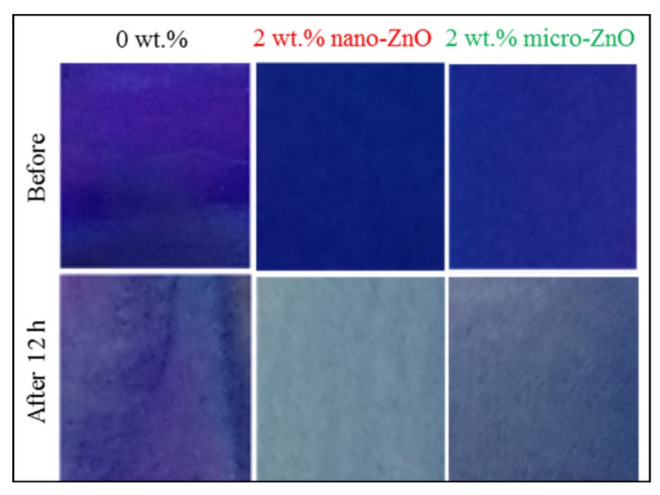
Photographs of methyl blue-contaminated coatings without or without ZnO particles, before and after 12 h of UV exposure.

**Figure 9:**
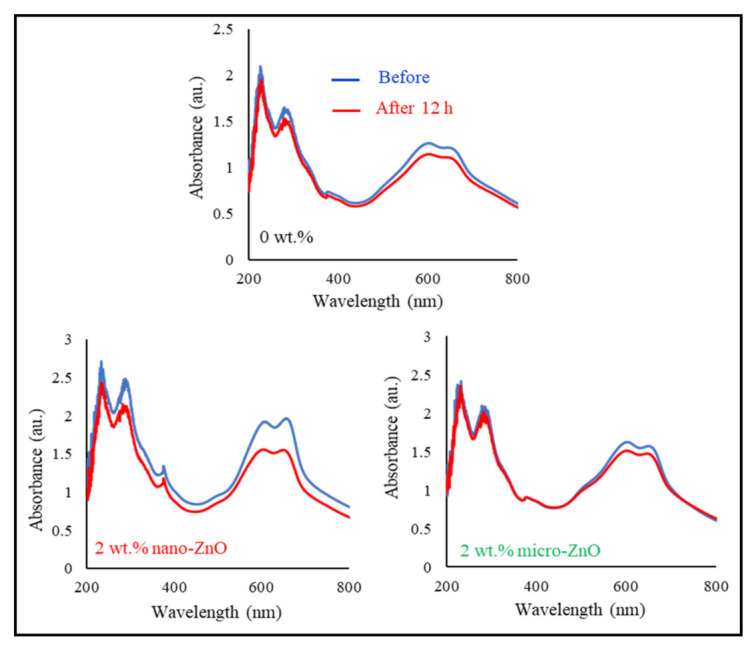
UV–vis spectra of the methyl blue-contaminated coatings with (2 wt.%) or without (0 wt.%) ZnO particles, before and after 12 h of UV exposure.

As an industrial dye, methylene blue has been commonly used as artificial dirt to evaluate the photocatalytic efficiency of metal oxides [5]. In the methylene blue removal (self-cleaning ability) test, each coating was irradiated by UV light for 12 h. Figure 8 and Figure 9 present photographs and UV–vis spectra of various coatings in the methylene blue removal test, respectively.

As shown in Figure 8, after 12 h of UV radiation exposure, the methylene blue was decolorized most efficiently by the nanocomposite coating and least efficiently by the neat coating. Similar results were obtained when investigating the UV–vis spectra (Figure 9). After being exposed to UV radiation for 12 h, the characteristic absorbance of methylene blue in the region of 500–700 nm decreased most strongly in the case of the coating containing 2 wt.% nano-ZnO and most weakly regarding the neat coating.

The fast decolorization and reduction of the characteristic UV absorbance of methylene blue in the case of the coating containing 2 wt.% nano-ZnO can be explained by the photocatalytic effect of the nanoparticles that led to the low adhesion of this dye. Hence, the dirt was easily separated from the coating. On the other hand, the photolysis may result in the decomposition of methylene blue as reported in the literature [37,38,39,40].

The obtained results experimentally proved that the photocatalytic activity of nano-ZnO was significantly stronger than that of micro-ZnO.

## 4. Conclusions

The effect of micro-/nano-ZnO particles on the properties of water-borne acrylic coatings was systematically investigated. The results showed that the incorporation of ZnO particles increased both the abrasion resistance and the methylene blue removal efficiency of the water-borne acrylic coatings, with nano-ZnO particles being the best. The dispersion of nanoparticles through sonication showed a complete dispersibility, with no polymer chain destruction observed in IR and UV–vis spectra. However, the weathering degradation of nanocomposite coatings was more severe as compared to the coating with micro-ZnO. Our finding indicates the promising use of nano-ZnO in multifunctional water-borne acrylic coatings.

## Data Availability

The data presented in this study are available on request from the corresponding author.

## Supplementary Materials

The following are available online at https://www.mdpi.com/2073-4360/13/5/717/s1, Figure S1: SEM micrograph of the nano-ZnO particles at different magnifications.
