# Supplementary material for: Water-Borne ZnO/Acrylic Nanocoating: Fabrication, Characterization, and Properties
Source: Polymers (Basel). 2021 Feb 26;13(5):717. doi: 10.3390/polym13050717 (PMC7956375; doi:10.3390/polym13050717)
Supplement: Supplementary file 1 [file polymers-13-00717-s001.zip › polymers-1121169-supplementary.pdf]

Water-Borne ZnO/Acrylic Nanocoating: Fabrication, Characterization, and Properties

Tien Viet Vu [1], Thien Vuong Nguyen [2,3,*], Mohammad Tabish [4], Sehrish Ibrahim [5], Thi Huong Thuy Hoang [6], Ram K. Gupta [7,*], Thi My Linh Dang [2], Tuan Anh Nguyen [2,*] and Ghulam Yasin [4,8,*]

[1] Faculty of Chemical Technology, Hanoi University of Industry, BacTuLiem, Hanoi 122300, Vietnam; vuviet.tphn@gmail.com
[2] Institute for Tropical Technology, Vietnam Academy of Science and Technology, Hanoi 122300, Vietnam; dangthimylinh.lhda58@gmail.com
[3] Graduate University of Science and Technology, Vietnam Academy of Science and Technology, 18 Hoang Quoc Viet, CauGiay, Hanoi 122300, Vietnam
[4] State Key Laboratory of Chemical Resource Engineering, College of Materials Science and Engineering, Beijing University of Chemical Technology, Beijing 100029, China; tabish.5000@buct.edu.cn
[5] Department of Zoology, University of Education, Lahore, Punjab 54770, Pakistan; sehrish.ibrahim@yahoo.com
[6] Hong Duc University, 565 Quang Trung, Dong Ve, Thanh Hoa 4000, Thanh Hoa Province, Vietnam; hhthuyhd77@gmail.com
[7] Department of Chemistry, Kansas Polymer Research Center, Pittsburg State University, Pittsburg, KS 66762, USA
[8] Institute for Advanced Study, College of Physics and Optoelectronic Engineering, Shenzhen University, Shenzhen, Guangdong 518060, China
* Correspondence: vuongvast@gmail.com (T.V.N.); ramguptamsu@gmail.com (R.K.G.); ntanh@itt.vast.vn (T.A.N.); yasin@mail.buct.edu.cn (G.Y.)

Mechanism of the decomposition of methylene blue

The radicals and intermediates such as $^{\bullet}OH$ and $O_2^{\bullet}$ (as mentioned in the introduction) play an important role in the photolysis mechanism of organic compounds. According to Feng et al, [1] the $^{\bullet}OH$ radical is the main oxidizing agent for the decomposition of methylene blue and other intermediates. Since methylene blue is a cationic dye, it is unable to donor electron. The initiation stage is the broken of $C\text{-}S^{+}\text{=}C$ bond of methylene blue:

$$R\text{-}S^{+}\text{=}R' + {}^{\bullet}OH \rightarrow R\text{-}S(\text{=}O)\text{-}R' + H^{+} \qquad \text{---(1)}$$

The second $^{\bullet}OH$ radical continuously attacks the sulfoxide radical to form sulfone compound, dividing 2 benzene rings by reactions (2) and (3):

$$NH_2\text{-}C_6H_3(R)\text{-}S(\text{=}O)\text{-}C_6H_4\text{-}R + {}^{\bullet}OH \rightarrow NH_2\text{-}C_6H_3(R)\text{-}SO_2 + C_6H_5\text{-}R \qquad \text{---(2)}$$

Or NH_2-C_6H_3(R)-S(=O)-C_6H_4-R + $^{\bullet}OH \rightarrow$ NH_2-C_6H_4-R + SO_2-C_6H_4-R ---(3)

After formed, the sulfone compound instantly reacts with the third $^{\bullet}OH$ radical to generate sulfonic acid:

$$SO_2\text{-}C_6H_4\text{-}R + {}^{\bullet}OH \rightarrow R\text{-}C_6H_4\text{-}SO_3H \quad \text{---(4)}$$

Finally, the fourth $^{\bullet}OH$ radical will attack sulfonic acid to release sulfate ion:

$$R\text{-}C_6H_4\text{-}SO_3H + {}^{\bullet}OH \rightarrow R\text{-}C_6H_4^{\bullet} + SO_4^{2-} + 2H^{+} \quad \text{---(5)}$$

Besides, the $^{\bullet}OH$ radical can replace the amine group in the methylene blue molecule to create phenol and let out $NH_2^{\bullet}$ radical:

$$R\text{-}C_6H_4\text{-}NH_2 + {}^{\bullet}OH \rightarrow R\text{-}C_6H_4\text{-}OH + NH_2^{\bullet} \quad \text{---(6)}$$

$$NH_2^{\bullet} + H^{\bullet} \rightarrow NH_3 \quad \text{---(7)}$$

$$NH_3 + H^{+} \rightarrow NH_4^{+} \quad \text{---(8)}$$

The two symmetric dimethyl-phenyl-amino groups of methylene blue are attacked by $^{\bullet}OH$ radical, generating ancol, aldehyde, the acid in order. Finally, the leaving carboxyl group is oxidized to form CO_2 according to the Kolbe photo reaction [1].

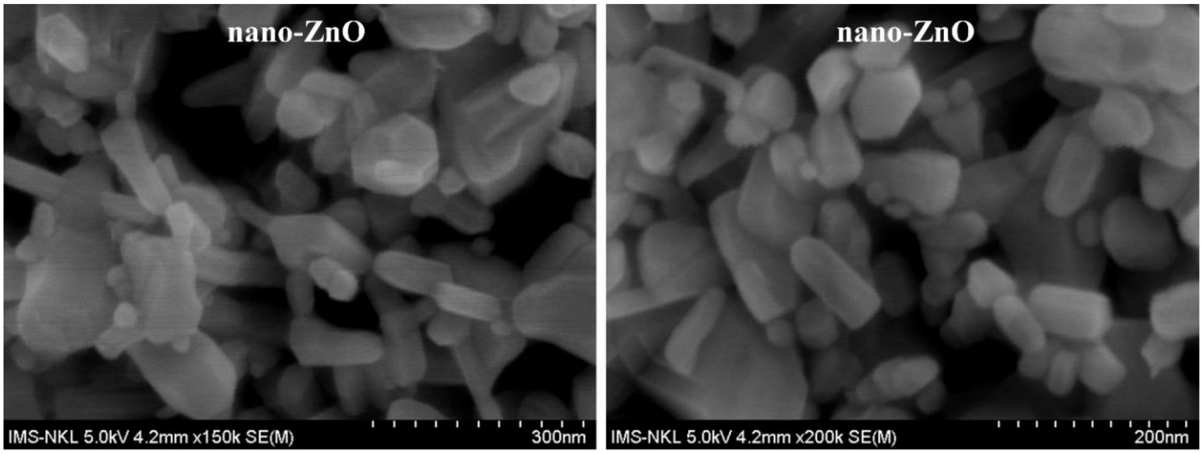

Figure S1. SEM micrograph of the nano-ZnO particles at different magnifications.

Reference

[1] F.P Feng, Z. Zhuo, Peng Peng, and D.X Gang, Photodegradation of Methylene Blue in a Batch Fixed bed Photoreactor Using Activated Carbon fibers supported TiO_2 photocatalyst, The Chinese J. Proc. Eng., 2008, 1(8), 65-71.
